# Supplementary figures and images for: Vitiligo and anxiety: A systematic review and meta-analysis
Source: PLoS One. 2020 Nov 10;15(11):e0241445. doi: 10.1371/journal.pone.0241445 (PMC7654800; doi:10.1371/journal.pone.0241445)

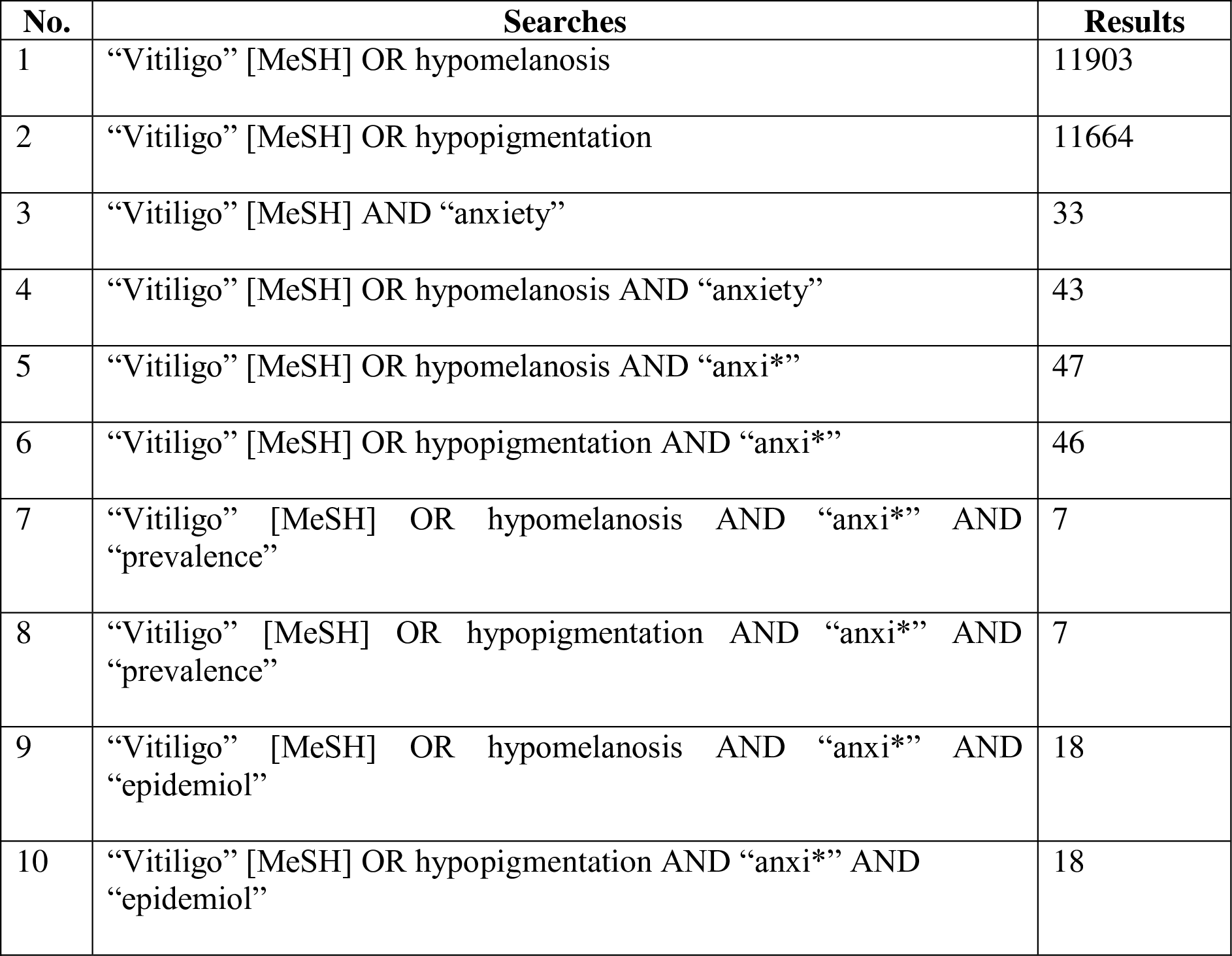

Supplement: S1 Table — (TIF) [file pone.0241445.s002.tif]

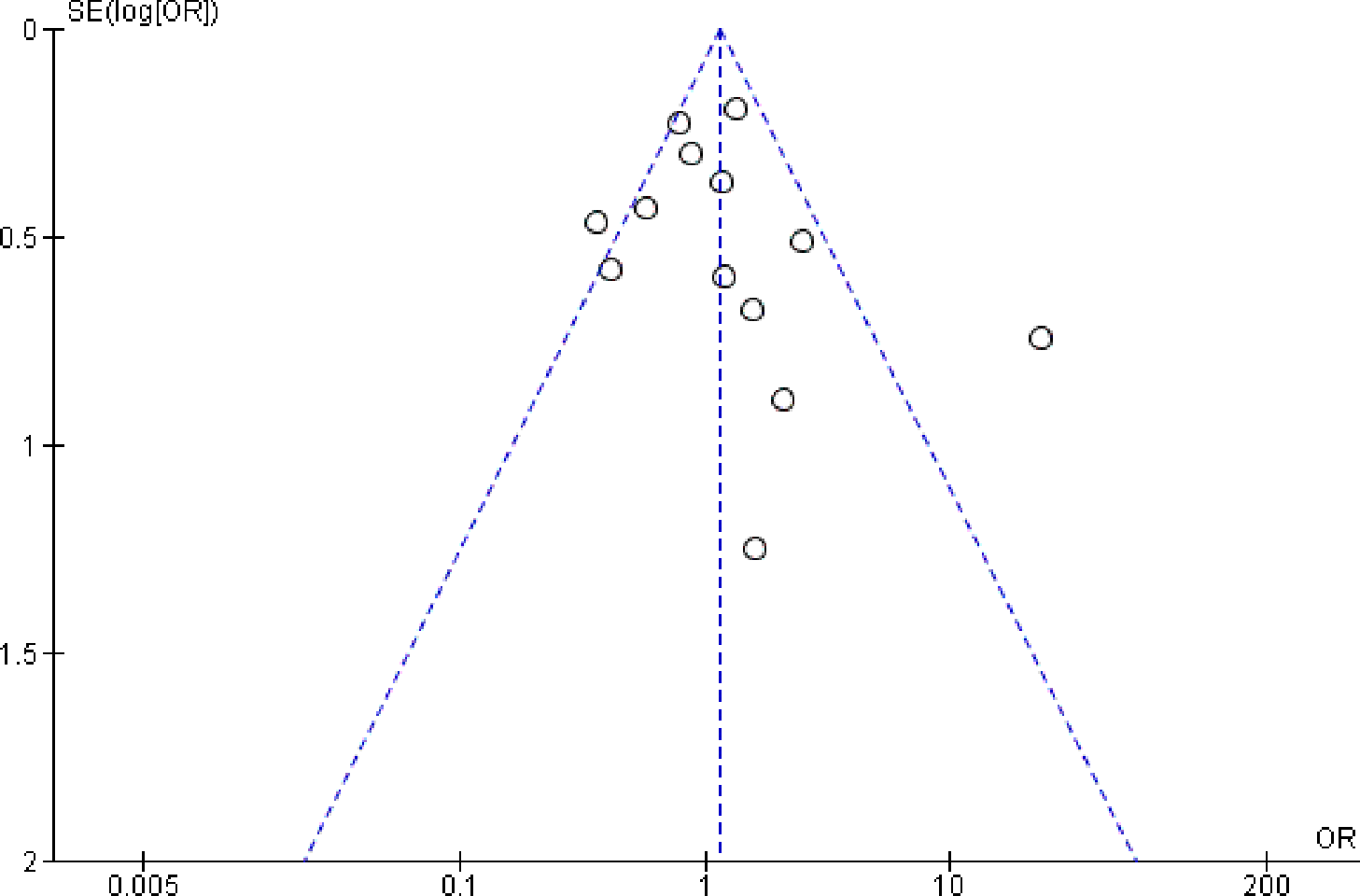

Supplement: S1 Fig — SE, standard error (Log[OR]); OR, odds ratio. (TIF) [file pone.0241445.s003.tif]

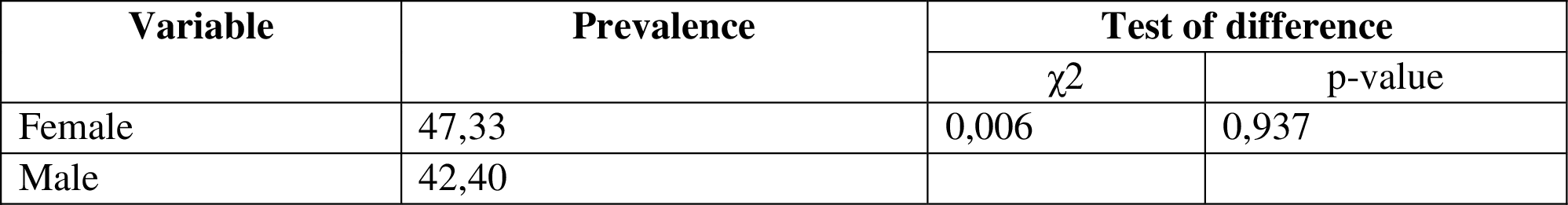

Supplement: S2 Fig — (TIF) [file pone.0241445.s004.tif]

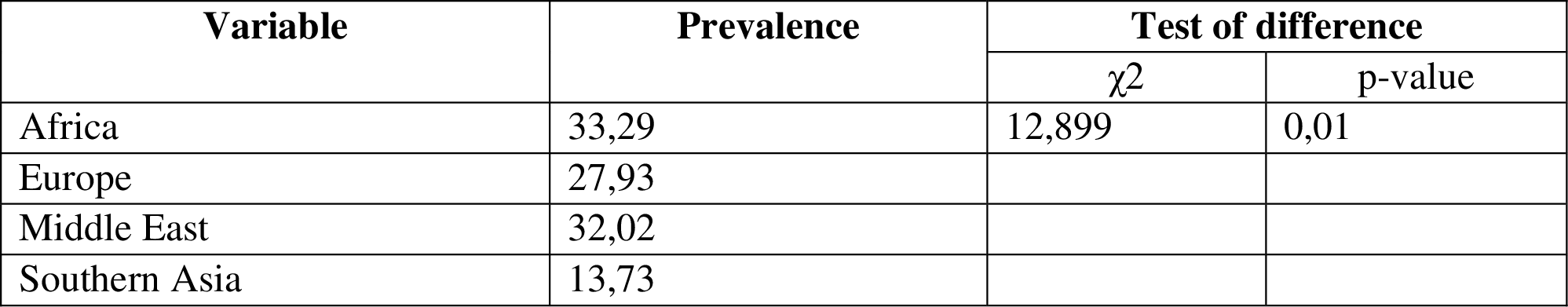

Supplement: S3 Fig — (TIF) [file pone.0241445.s005.tif]

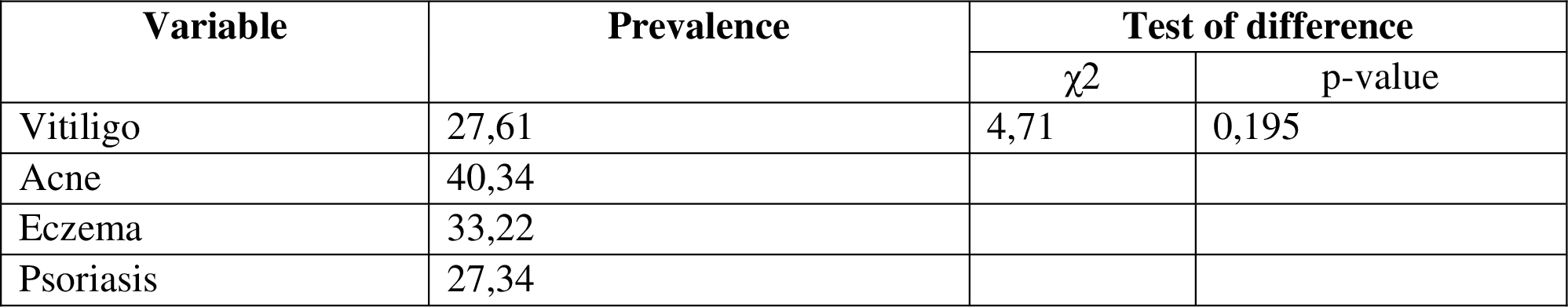

Supplement: S4 Fig — (TIF) [file pone.0241445.s006.tif]

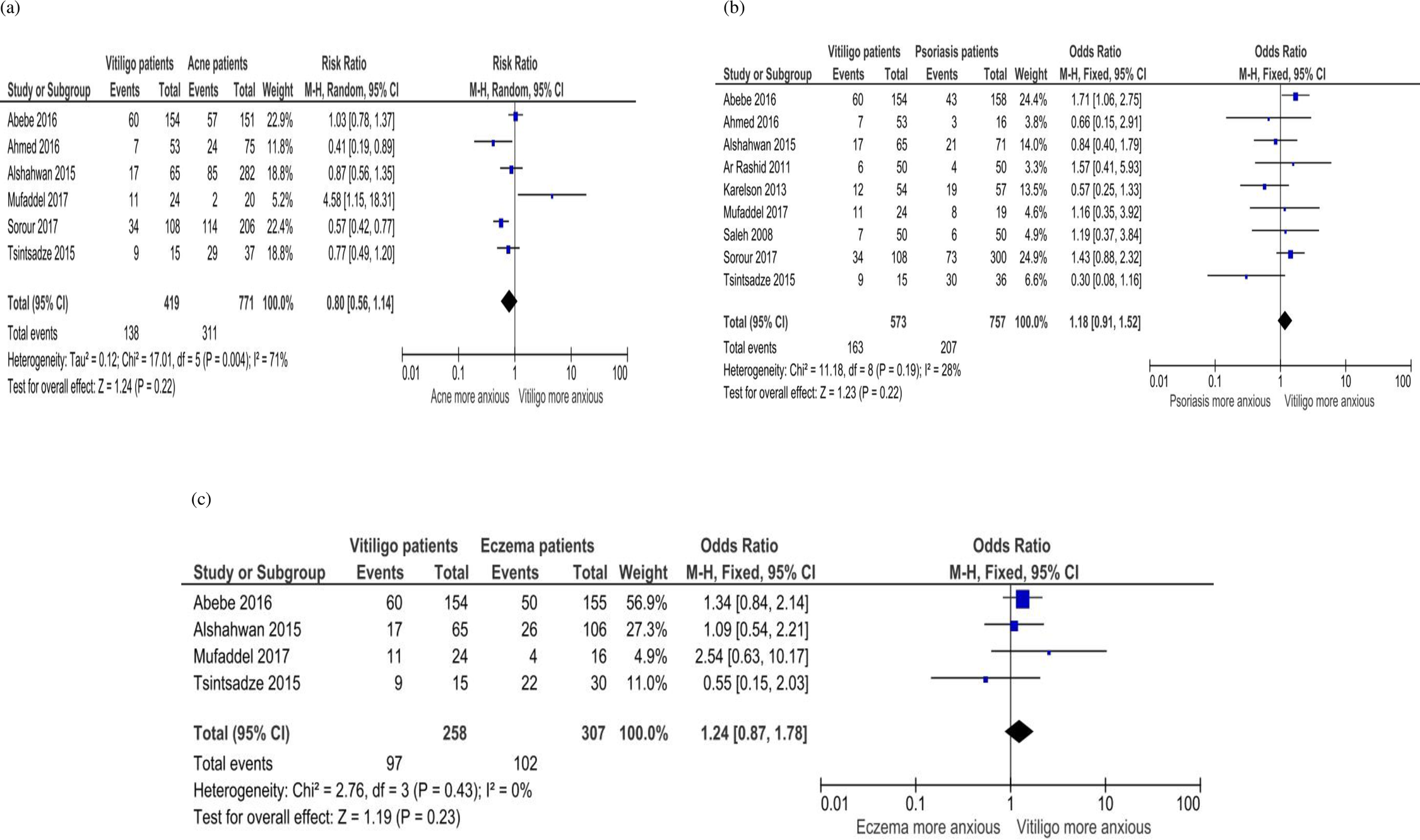

Supplement: S5 Fig — Meta-analysis of the prevalence of anxiety in patients with vitiligo compared with those with acne (a), psoriasis (b), eczema (c). (95% CI: = 95% Confidence Interval; M-H, Mantel-Haenszel Method. (TIF) [file pone.0241445.s007.tif]

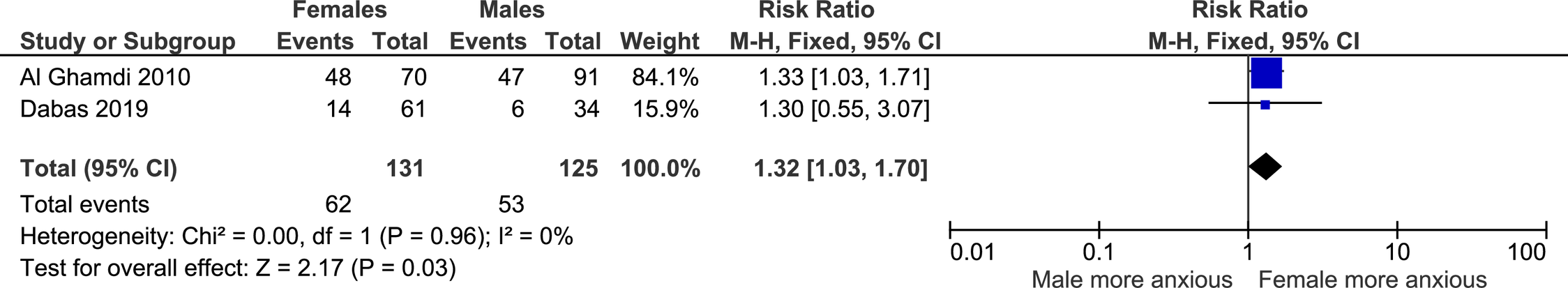

Supplement: S6 Fig — (95% CI: = 95% Confidence Interval; M-H, Mantel-Haenszel Method). (TIF) [file pone.0241445.s008.tif]

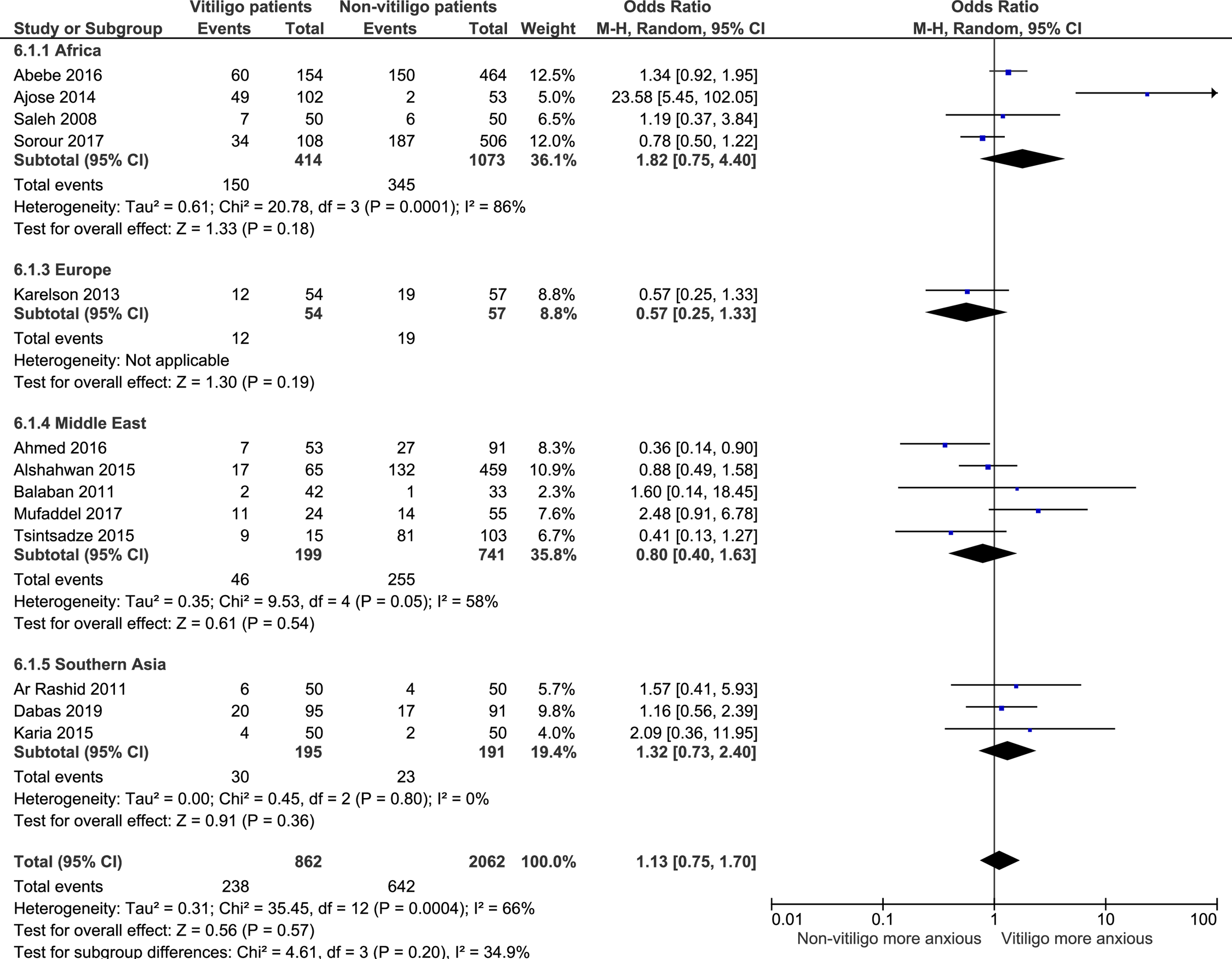

Supplement: S7 Fig — (Сhi2, Chi-Squared Test; df, degrees of freedom; Z, statistical test; I2, meta-analysis heterogeneity index; P < 0,05 (two-tailed)). (TIF) [file pone.0241445.s009.tif]
