# Supplementary material for: Vitiligo and anxiety: A systematic review and meta-analysis
Source: PLoS One. 2020 Nov 10;15(11):e0241445. doi: 10.1371/journal.pone.0241445 (PMC7654800; doi:10.1371/journal.pone.0241445)
Supplement: Attachment — Submitted filename: Response to Reviewers.docx [file pone.0241445.s010.docx]

# Response to Reviewers:

# Journal: PLoS ONE

**Manuscript No:**

**Manuscript title:** Vitiligo and Anxiety: A Systematic Review and Meta-Analysis

**Authors:**	Kussainova et al.

**Additional Editor Comments:**

While some scientists occasionally have considered the systematic review (SR) as the top level of evidence for clinical science research in the past, please note that SR per se cannot be endorsed solely due to their format ("because they are a systematic review"), but must be critically evaluated for the problem reviewed in the respective article. Consequently, different qualities of RCTs (as given with your study, please see below), explicit statements of protocols, careful estimates of risk bias, large numbers of studies, and sophisticated statistical meta-analyses are not considered true substitutes for a thorough understanding of the literature, for critical analysis, and for scientifically sound and clinically relevant conclusions considered generalizable. The authors might additionally wish to go to R. Solow, Systematic review versus structured critical analysis, Cranio 1-13 (2019). <https://doi.org/10.1080/08869634.2019.1614288>, and discuss these problems. Thank you very much for considering our manuscript for publication. We treated all comments with all due respect and did our best to respond to the reviewers comments.

Subgroup analyses would seem necessary, as well as analysis of heterogeneity. Please note that the latter is being consistently underestimated in meta-analyses, and one measure of heterogeneity is I², thus indicating the percentage of variance in your meta-analysis. Thank you. We introduced the heterogeneity analysis.

A major concern would be the quality of included papers. Please remember that the quality of a randomized trial, or, as it has been shown in monthly practice of publishing, the lack thereof, is not dependent on who is evaluating this trial. It is the trial is the trial is the trial, and, again, it is - the trial. In other words, some scientists would say: "If you put garbage in, there will be garbage out." With this in mind, the reviewer does not agree to "finally rely on studies with quality scores of 50% and above that will be included in this study". We strongly agree with this statement and thus, excluded three poor quality studies from our meta-analysis.

Please note that there is a public trust that research will be conducted to the highest standards possible. The main drawback of the current submission, would seem that the standards discussed above might have been disregarded. Significant problems have been found with previous systematic reviews, thus questioning the reliability of the latter as the premier level of evidence for clinical science research. Just being aware of those deficiencies and drawbacks does not help - correct interpretation of valid clinical research should be done by the scientist, and not by the reader alone (even if the latter must always be alert). All other scenarios will lead wasting resources. Thank you. Done. Please, see the changes listed below to evaluate the work done in this regard.

We are grateful for your personnel recommendations. It helped us to improve quality of our manuscript. Also revision process allowed us to find some technical mistakes which are successfully corrected (e.g. reference list in Manuscript).

| **Journal requirements** | **Changes by the authors** |
| --- | --- |
| Please ensure that your manuscript meets PLOS ONE's style requirements, including those for file naming. The PLOS ONE style templates can be found at https://journals.plos.org/plosone/s/file?id=wjVg/PLOSOne_formatting_sample_main_body.pdf and  https://journals.plos.org/plosone/s/file?id=ba62/PLOSOne_formatting_sample_title_authors_affiliations.pdf | Thank you for considering our manuscript. We have addressed all the proposed amendments. |
| At this time, we ask that you please provide the full search strategy and search terms for at least one database used as Supplementary Information. | Thank you. Done. Search Strategy Pubmed (see S1 Table) No. \| Searches \| Results; 1 \| “Vitiligo” [MeSH] OR hypomelanosis \| 11903; 2 \| “Vitiligo” [MeSH] OR hypopigmentation \| 11664; 3 \| “Vitiligo” [MeSH] AND “anxiety” \| 33; 4 \| “Vitiligo” [MeSH] OR hypomelanosis AND “anxiety” \| 43; 5 \| “Vitiligo” [MeSH] OR hypomelanosis AND “anxi*” \| 47; 6 \| “Vitiligo” [MeSH] OR hypopigmentation AND “anxi*” \| 46; 7 \| “Vitiligo” [MeSH] OR hypomelanosis AND “anxi*” AND “prevalence” \| 7; 8 \| “Vitiligo” [MeSH] OR hypopigmentation AND “anxi*” AND “prevalence” \| 7; 9 \| “Vitiligo” [MeSH] OR hypomelanosis AND “anxi*” AND “epidemiol” \| 18; 10 \| “Vitiligo” [MeSH] OR hypopigmentation AND “anxi*” AND “epidemiol” \| 18 Search strategy for other databases includes utilizing of key words with subsequent selection of published manuscripts based on their titles and abstracts. Unfortunately, the use of Bullean operators and additional filters was not possible in above mentioned data bases. |
| Please include the methodology and Results of any analyses conducted for the assessment of publication bias by Begg's funnel plot or and/or Egger's regression plot in your meta-analysis. | We evaluated publication bias using the Egger’s graphical method (Egger, M.; Davey Smith, G.; Schneider, M.; Minder, C. Egger, M.; Davey Smith, G.; Schneider, M.; Minder, C. Bias in meta-analysis detected by a simple, graphical test. BMJ 1997, 315, 629–634.)  S2 Fig represents the funnel plot of comparison: Anxiety in vitiligo and non-vitiligo patients, outcome: Anxiety. Almost all studies (more than 95%) fall in symmetrical distribution that confirms minimal risks of selection bias. Thus, the funnel plot did not suggest any publication bias (see S2 Fig).  S2 Fig. Funnel plots of studies evaluating the comparison: anxiety in vitiligo and non-vitiligo patients. SE, standard error (Log[OR]); OR, odds ratio. |
| Please amend your list of authors on the manuscript to ensure that each author is correctly linked to each affiliation. Authors’ affiliations should reflect the institution where the work was done (if authors moved subsequently, you can also list the new affiliation stating “current affiliation:….” as necessary). | Thank you. Done. |

| **Reviewer 1** | **Changes by the authors** |
| --- | --- |
| Abstract  - "Statistically significant difference in anxiety rates was found among female and male patients (34.46% vs 32.78%)  (P =0.04)." Do the authors think that this difference would be clinically relevant?  - "The pooled odds ratio among vitiligo and non-vitiligo patients indicated a statistical significance in African countries (1.39 [1.07, 1.79]; P =0.01)." Do the authors think that this would be astonishing? | Thank you very much for reviewing our manuscript. Your comments helped us to improve it significantly.  We highlighted all changes made in yellow  We excluded three articles from our systematic review due to their poor-quality, so some statistical findings have also been changed.  Statistically significant difference in anxiety rates was found among female and male patients (~~34.46% vs 32.78%~~ 47.32% vs 42.4%) (*~~P~~*~~=0.04~~ *P*=0.03), but the clinical relevance of this issue remains arguable.  Also,  The pooled odds ratio among vitiligo and non-vitiligo patients did not indicate a statistical significance among different continents. |
| Intro  - "Unlike depression, anxiety among patients with vitiligo is less studied in the current literature." This alone would not justify any research on this topic.  - Althors have failed to convincingly elaborate both aims and objectives.  - A clear and indisputable null hypothesis is missing. remember that H0 must be deducible from the foregoing thoughts. | Thank you. You're right. This statement was replaced by the following:  Although the sufficient number of papers devoted to the issue of anxiety in vitiligo patients had been published before, there is no pooled evidence that is needed for comprehensive understanding of this problem.  To overcome this drawback, we added this information:  The specific goals of the present systematic review and meta-analysis are:   - To determine the prevalence (or event rate) of anxiety in vitiligo patients in comparison with non-vitiligo patients. - To investigate the impact of some variables such as gender, continent, type of skin disorder on anxiety rate among vitiligo and non-vitiligo patients.   In this systematic review the null hypothesis is that there is no difference in prevalence of anxiety among vitiligo and non-vitiligo persons. |
| Results  - Please compare "Unlike depression, anxiety among patients with vitiligo is less studied in the current literature." with "Finally, 18 articles with 1419 cases of vitiligo were included to our study." Do you really think that this is a "less studied" context? This reviewer would not agree.  - "Thus, our meta-analysis includes 18 studies comprising 1419 vitiligo patients (...)." See above. Why do you repeat this information?  - Including cross sectional studies would seem difficult with respect to a valid outcome.  - "Evaluating the quality of included studies with Newcastle-Ottawa scale, we found that eight studies met the criteria of fair quality, the same number of studies was found to be of good quality, and two studies were of poor quality." This is considered too less quality for a valid conclusion. Please note that some scientists occasionally have considered the systematic review (SR) as the top level of evidence for clinical science research in the past. However, you should remember that SR per se cannot be endorsed solely due to their format ("because they are a systematic review"), and must be critically evaluated for the problem reviewed in the respective article. Consequently, different qualities of RCTs, explicit statements of protocols, careful estimates of risk bias, large numbers of studies, and sophisticated statistical meta-analyses are not considered true substitutes for a thorough understanding of the literature, for critical analysis, and for scientifically sound and clinically relevant conclusions. | Thank you. You're right. We have removed this sentence from the text.  Thank you. You're right. We have removed this sentence from the text.  As the aim of our systematic review was to identify any differences in anxiety rate among vitiligo and non vitiligo patients, the cross sectional studies were considered by us as relevant (or suitable) enough to extract the needed epidemiological data, in particular, the prevalence or event rate.  Thank you. You're right. Based on your recommendation, we excluded studies that were rated as "poor" on the Newcastle-Ottawa scale. This recommendation improved significantly the quality of our research. We reflected this in exclusion criteria statement:  Exclusion Criteria  Our exclusion criteria were as follows: (i) studies that did not state the rate of anxiety among vitiligo patients or provided insufficient data for calculation of anxiety rates; (ii) unavailability of the full text for full review; (iii) studies with low methodological quality, i.e. case reports, case series and commentaries; (iv) studies published in other languages apart from English and (v) studies that were considered to be of low-quality by Newcastle-Ottawa scale (˂4 points). |
| Disc  - "We demonstrated that patients with vitiligo do not have a significantly higher risk of anxiety or anxiety symptoms as compared to those who present with no pigmentary skin disorders." Again, this would seem confirmative only, and is considered expectable. Thus, let me repeat my question: What rationale triggered your study?  - "This limitation derives from available publications on the study topic as they are often imprecise in identifying what is (...)." This reviewer would like to agree. Some researchers would say "if you put garbage in, you will get garbage out". In other words, if the quality of included studies, is low, there will be a poor output only. However, in this case, doing such a research would seem doubtful. | Thank you. We have added this information:  We expected that patients with vitiligo have a significantly higher risk of anxiety or anxiety symptoms as compared to those who present with no pigmentary skin disorders. In fact, after conducted systematic review and meta-analysis the null hypothesis rejecting any difference in prevalence rates of anxiety among comparison groups was reconfirmed.  Thank you. You're right. Based on your recommendation, we excluded studies that were rated as "poor" on the Newcastle-Ottawa scale, as it was mentioned previously. |
| Concl  - Please do not simply repeat your results here. Instead, provide a reasonable extension of your outcome. | Done. We totally revised this part of the manuscript:  In conclusion, the patients with vitiligo suffer from anxiety as frequently as do individuals with such severe skin conditions, as psoriasis or eczema. Although vitiligo patients present with no symptoms apart from decolorated skin patches, this pathology is accompanied with various psychological problems. Dermatologists and other specialists dealing with vitiligo patients should be aware of their predisposition to anxiety and be able to envisage correctional interventions to alleviate the burden of their mental distress. Clinical guidelines have to contain information about effective screening and management of anxiety among vitiligo patients. Finally, we did not find statistically significant impact of various risk factors on anxiety rate. This fact emphasizes the global burden of vitiligo that is not dependent on patient’s gender or ethnicity. |
| Refs  - Please check Guidelines for Authors, and check uniform formatting. | Thank you. Done. |

| **Reviewer 2** | **Changes by the authors** |
| --- | --- |
| I appreciate your work, but the following data and analyzes are missing, and the following questions are open.  For all vitiligo patients i2 in meta-analysis | Thank you for your kind comments. They helped us to improve the quality of our manuscript.  We highlighted all changes made in yellow  We excluded three articles from our systematic review due to their poor-quality, so some statistical findings have been changed.  I2 = 66 % for all vitiligo patients i2 in meta-analysis  There was moderate heterogeneity between the studies ~~(I~~^~~2~~^~~=62%;~~ *~~P~~*~~= 0.0008)~~ (I^2^=66%; *P*= 0.0004). |
| For subgroups  Continents (prevalence and p-value for each continent)  Gender (prevalence and p-value for females and males)  Skin disorders (prevalence and p-value for the skin disorders you mentioned) | We add additional information on subgroup analyses to the text.  Also, we performed the subgroup analysis of anxiety rate among vitiligo patients according to their gender, type of skin disorder and continent of residence. The statistically significant difference in prevalence rates was found comparing African, European, Middle East and South Asian countries (33.29%, 27.93%, 32.02%, 13.73% respectively; P= 0.01 using χ2- statistics) (see S3 Fig.). However, there was no difference in prevalence rates based on patient gender and type of skin disorder in the subgroup analyses (see S4 Fig. and S5 Fig.). |
| We used the random-effects model when heterogeneity 60% across the studies was  112 large (i2> 60%, P< 0.05) and fixed-effects meta-analysis at small heterogeneity (i2< 60%, P< 0.05).  Did you have a reference for this statement?  Is it not right to use random-effects model in your study? | I2 statistic is the test used to quantify heterogeneity and calculates the proportion of variation due to heterogeneity rather than due to chance. The I2 value ranges from 0% to 100%, with higher values indicating greater heterogeneity. As a rough guide, the I2 statistic can be interpreted as follows:  • 0% to 40%: might not be important;  • 30% to 60%: may represent moderate heterogeneity*  • 50% to 90%: may represent substantial heterogeneity* • 75% to 100%: considerable heterogeneity* (<https://cccrg.cochrane.org/sites/cccrg.cochrane.org/files/public/uploads/heterogeneity_subgroup_analyses_revising_december_1st_2016.pdf>).  In our study, we assessed the range from 0 to 60% as a small heterogeneity, while other share as a large heterogeneity.  We have used THE random-effects model for meta-analysis as this includes consideration of heterogeneity in the effect estimate (heterogeneity = i2 > 60%, P< 0.05).  A fixed-effect model assumes that there is no statistical heterogeneity between the studies.  When studies are gathered from the published literature, the random-effects model is generally a more plausible match (<https://www.meta-analysis.com/downloads/Meta-analysis%20Fixed-effect%20vs%20Random-effects%20models.pdf>) |
| 1. How do you definite the event rate?  2. What is your aim to report the event rate?  The formula?    3. Is it not more logical to report the rate in a cross-sectional study? | 1. Event rate is the proportion of people with a specific condition in a group in whom the event is observed.    2. We aimed to report the event rate of anxiety to give comprehensive characteristics of total sample and the study group  Formula:  a) Event rate of anxiety in total sample = total number of cases with anxiety/total number of sample  b) Event rate of anxiety in vitiligo patients = total number of vitiligo patients with anxiety/total of vitiligo patients  3. Taken into account that this meta-analysis was objected to study epidemiological features of anxiety in vitiligo patients, we used both ‘Event rate’ and ‘Prevalence’ to characterize total population. |

| **Reviewer 3** | **Changes by the authors** |
| --- | --- |
| It's a good quality systematic review. The main limitation of this study was the different screening tools used in the primary studies to identify anxiety symptoms.  So I recommend to explain in the manuscript if you tried to do a subgroup analysis according to the anxiety scales apart from the meta-analysis for gender, control group and geographic regions.  I also suggest to mention if you tried to do a meta-analysis acoording to the extent of vitiligo and the prevalence of anxiety symptoms. | Thank you very much for your comments, they really helped us to improve the quality of our manuscript.  We highlighted all changes made in yellow:  We have added this information to the text of our manuscript:  Also, the wide range of anxiety assessment tools did not allow us to perform the subgroup analysis according to this criterion.  Besides, due to the lack of data on prevalence of anxiety symptoms according to severity of vitiligo it was impossible to conduct the sub-analysis of anxiety rate regarding to extent of depigmentation. |
