# Supplementary material for: Vitiligo and anxiety: A systematic review and meta-analysis
Source: PLoS One. 2020 Nov 10;15(11):e0241445. doi: 10.1371/journal.pone.0241445 (PMC7654800; doi:10.1371/journal.pone.0241445)
Supplement: Attachment — Submitted filename: Response to Reviewers.docx [file pone.0241445.s011.docx]

# Response to Reviewers:

# Journal: PLoS ONE

**Manuscript No:**

**Manuscript title:** Vitiligo and Anxiety: A Systematic Review and Meta-Analysis

**Authors:**	Kussainova et al.

| **Reviewer 1** | **Changes by the authors** |
| --- | --- |
| This resubmitted draft has considerably improved with the first round of revisions. However, the current version still would not seem convincing, and is not considered ready to proceed.  Abstract  - Contents would seem confusing. See aims: "We aimed to evaluate the prevalence of anxiety among patients with vitiligo and to elucidate the associated risk factors." Thus, anxiety and vitiligo was searched for. However, suddenly, other information will appear: "the anxiety rate for acne patients was higher than for vitiligo, eczema and psoriasis patients". Please revise thoroughly.  - "The pooled odds ratio among vitiligo and non-vitiligo patients did not indicate a statistical significance among different continents." Did you aim to find differences between continents. I guess you did not. Please revise.  - "In addition, the anxiety rate for acne patients was higher than for vitiligo, eczema and psoriasis patients, but it was not statistically significant." If was NOT statistically significant, would it be worth mentioning? Why would it be worth mentioning, if these differences would be by chance? Please revise.  - "This finding accentuates the necessity of anxiety awareness in management of vitiligo patients." There were no differences, right? And anxiety has no influence, right? So, why would "the necessity of anxiety awareness in management of vitiligo patients be accentuated"?  - In total, this section would not seem convincing. Please note that Abstract section and full text obviously do not correspond. | Thank you very much for re-reviewing our manuscript. You have done a great job and we will try to give detailed answers to all comments.  We highlighted all changes made in yellow  Thank you. Done. We have paraphrased the aim of our study  We aimed to evaluate the prevalence of anxiety among patients with vitiligo from different countries and to compare it with patients suffering from eczema, psoriasis, and acne. ~~and to elucidate the associated risk factors~~.  Done. We made this statement more concrete:  In addition, the pooled odds ratio among vitiligo and non-vitiligo patients did not indicate a statistical significance among patients coming from different continents.  Thank you. We have removed this sentence from the text.  Thank you. We have paraphrased this part of the conclusion:  This finding accentuates the necessity of anxiety awareness in management of ~~vitiligo~~ patients with skin diseases.  Thank you. The abstract was revised according to your recommendations. |
| Intro  - "Globally, there is an increasing rate of anxiety disorders (...)." I would have some doubts on that postulated increase. Please compare to "The rate of anxiety disorder in a general population is around 12% (...)" in the same paragraph. This would seem contradicting.  - "In certain cultures individuals suffering from vitiligo may be stigmatized and could experience difficulty with finding a couple or staying employed." Reference missing. remember that each statement must be underlined with a reference.  - "Although the sufficient number (...)." What is "the" sufficient number?  - "Such, the existing data indicate that patients with vitiligo face a higher risk of mental distress, although the current evidence coming from pooled analyses is insufficient." How can you say that - if there is no evidence - patients with vitiligo would face a higher risk? This would not seem convincing.  - "A 2017 systematic review and meta-analysis on the prevalence of depression among patients with vitiligo estimated that the pooled prevalence of depression was 0.253 across 25 studies [9]. A 2018 meta-analysis of the prevalence and odds of depression in patients with vitiligo found a wide range of prevalence between 8% and 33% across 17 studies, depending on the diagnostic tool used. [10]." Please elaborate clearly what would be new with your study. From Table 1, it seems clear that you have included only one 2019 paper.  - Please revise for grammar and style. Must read "the null hypothesis was (...)."  - Again, please compare your Abstract section: The aims given in the Intro section do not correspond to your Results given in the Abstract section. | Thank you. We have removed this sentence from the text.  ~~The rate of anxiety disorder in a general population is around 12% and certain categories of people are predisposed to it to a higher extent [5].~~  Thank you. We added the reference after this statement.  39. Porter JR, Beuf AH, Lerner A, Nordlund J. Psychosocial effect of vitiligo: A comparison of vitiligo patients with “normal” control subjects, with psoriasis patients, and with patients with other pigmentary disorders. J Am Acad Dermatol. 1986;15(2):220–224. doi:10.1016/s0190-9622(86)70160-6.  Thank you. We have paraphrased this sentence.  Although the ~~sufficient number~~ relatively many papers devoted to the issue of anxiety in vitiligo patients had been published before, there is no pooled evidence that is needed for comprehensive understanding of this problem.  Thank you. We have paraphrased this sentence.  Such, the existing data indicate that patients with vitiligo possibly face a higher risk of mental distress, although the current evidence coming from pooled analyses is insufficient. In this systematic review the null hypothesis is that there is no difference in prevalence of anxiety among vitiligo and non-vitiligo persons.  Firstly, in above mentioned studies the authors aimed to find the prevalence of depression in vitiligo patients, while in our study we aimed to find the prevalence of anxiety.  Secondly, depression is not always associated with anxiety.  And finally, even one additional paper can impact significantly on the result of meta analysis.  Thank you. Done.  In this systematic review the null hypothesis ~~is~~ was that there is no difference in prevalence of anxiety among vitiligo and non-vitiligo persons.  Thank you. Done.  Therefore, the aim of this study was to evaluate the prevalence of anxiety among patients with vitiligo from different countries and to compare it with patients suffering from eczema, psoriasis, and acne ~~and to elucidate the associated risk factors~~ by conducting a systematic review and meta-analysis of published observational studies. |
| Meths  - Heterogeneity must read I².  - "(...) some studies contained the data on the prevalence of anxiety among patients with eczema, atopic dermatitis, (...)." But not all studies, right? However, how can you draw any conclusions from this? Again, please see Abstract section. | Thank you. Done.  The *~~i~~^~~2~~^* I^2^ value to assess heterogeneity among studies was processed. We used the random-effects model when heterogeneity across the studies was large (*~~i~~^~~2~~^* I^2^ > 60%, *P*< 0.05) and fixed-effects meta-analysis at small heterogeneity (*~~i~~^~~2~~^* I^2^ < 60%, *P*< 0.05).  Several studies involve patients with eczema, psoriasis, and acne as comparators for the vitiligo group. Subsequently, these skin diseases were included to subgroup analysis. Other studies contain the data on the prevalence of anxiety in other control groups (e.g. healthy controls), but the number of participants was not sufficient for a subgroup analysis. |
| Results  - "The prevalence of anxiety was 40.38% for acne patients vs 32.93% for vitiligo patients (...)." Again, this would seem confusing, since your aims did not explain other (non-vitiligo) skin diseases. Please revise thoroughly. | Thank you. We have paraphrased the aim of our study. |
| Disc  - Again, please see comments given above: "This meta-analysis aimed at evaluation of the prevalence of anxiety among vitiligo patients (...)."  - "In fact after conducted systematic review and meta-analysis the null hypothesis rejecting any difference in prevalence rates of anxiety among comparison groups was reconfirmed." Meaning would seem unclear, please revise.  - "(...) we found that acne patients have the highest risk of anxiety development." This was NOT your aim, right?  - Same with psoriasis and eczema, right?  - Same with depression, right? | Thank you. We have paraphrased the aim of our study.  This meta-analysis aimed to evaluate the prevalence of anxiety among patients with vitiligo from different countries and to compare it with patients suffering from eczema, psoriasis, and acne. ~~and to elucidate the associated risk factors~~.  Thank you. We have revised this sentence.  "In fact after conducted systematic review and meta-analysis the null hypothesis was reconfirmed, there was no difference ~~rejecting any difference~~ in prevalence rates of anxiety among comparison groups ~~was reconfirmed."~~  We have paraphrased the aim of our study.  We have paraphrased the aim of our study.  The aim of our study was to find the pooled prevalence of anxiety, but not the depression. |
| Refs  - Again, please revise for uniform formatting. Check tiles, and revise thoroughly.  In total, this revised draft would not seem ready to proceed. | Thank you. All references were drown up according to author instructions.  Thank you. We have revised our draft according to your recommendations. |

| **Reviewer 2** | **Changes by the authors** |
| --- | --- |
| Dear Authors,  1. I would suggest that in the manuscript, the authors uniformly use event rate or prevalence. Concerning the definition, the author provided for the event rate that is the same as prevalence; hence, it would be better to unify these terms in the whole manuscript.  2. Using a random effect is a correct solution in this manuscript, but the rationale incorrect. Using a fixed- or random-effect model based on the results of the heterogeneity test is a common mistake in the meta-analysis. The followings are the explanations regarding this important issue.  “Some researchers start the analysis by selecting the fixed-effect model. They then test-perform a statistical test for heterogeneity in effect sizes (the Q-test).  • If the test for heterogeneity is not statistically significant, they conclude that the fixed-effect model is consistent with the data, and use this model in the analysis.  • If the test for heterogeneity is statistically significant, they conclude that the fixed-effect model is not consistent with the data, and use the random-effects model in the analysis.  This approach is fundamentally flawed for two reasons.  Reason 1  If we want to choose a model based on the sampling frame, then we should select the model based on our understanding of how the studies sampled, and not the results of a statistical test. If we are working with studies that assess the impact of an intervention in different populations, then logic tells us that the random-effects model is the model that fits the data, and it’s the model that we should choose.  To suggest that a non-significant p-value justifies the use of a fixed-effect analysis is to recommend that the lack of significance proves that the null is correct (that the studies share a common effect size). As we all learned in our first statistics class, the lack of significance does not prove that the null is true. And here, logic tells us that the null is probably false.  Reason 2.  The “flawed” approach uses the fixed-effect model as the starting point and requires evidence (a significant test of heterogeneity) to shift to the random-effects model.  The random-effects model should be the logical starting point. The random-effects model says that the true effect size may or may not vary from study to study, and thus does not assume that either is the case. As part of the analysis, we estimate the amount of variance in true effects across studies, and the estimate may or may not be zero.  By contrast, the fixed-effect model requires that the true effect size does not vary from study to study. Therefore, the fixed-effect model is more restrictive. It imposes a constraint that is neither necessary nor plausible.  If we should be using the random-effects model and (by mistake) employ the fixed-effect model, then it’s likely that  • The estimate of the mean will be incorrect  • The standard error will be incorrect  • The test of significance for mean will be incorrect  • The confidence interval about the mean effect will be too narrow  More fundamentally, the choice of a model defines the goals of the analysis.  The choice of a model determines the meaning of the summary effect  • Under the fixed-effect model, there is only one true effect. The summary effect is an estimate of that value.  • Under the random-effects model, there is a distribution of true effects. The summary effect is an estimate of that distribution’s mean.  One of the most important goals of a meta-analysis is to determine how the effect size varies across studies.  • When we use the fixed-effect model, we can estimate the common effect size, but we cannot discuss how the effect size varies, since this model assumes that the true effect size is the same in all studies.  • By contrast, if we elect to work with the random-effects model, we can ask not only “What is the mean effect size” but also “How does the effect size vary across populations.” In many cases, this question is key to understanding the effectiveness of the intervention.”  Conclusion  In other words, the analyses are correct, but when there is the heterogeneity, is random effects applied.  Good luck | Thank you. Your comments were very valuable to us.  We highlighted all changes made in yellow  1. We have deleted event rate and describe only prevalence in the study.  To determine the prevalence ~~(or event rate)~~ of anxiety in vitiligo patients in comparison with non-vitiligo patients.  2. We assume that the studies included in our meta-analysis using random-effects model could be a random sample from the set of studies. Also, we assume that the results of each study included in the meta-analysis represent the effect size for a particular study, which may vary within the population mean effect size. [Cohn LD, Becker BJ. How meta-analysis increases statistical power. Psychol Methods. 2003;8:243-253. http://dx.doi. org/10.1037/1082-989X.8.3.243; Fleiss JL. The statistical basis of meta-analysis. Stat Methods Med Res. 1993;2:121-145.].  We can suggest that the results of each study in this meta-analysis represent a unique effect. Because of this assumption, larger studies are given proportionally less weight, and, vice versa the smaller studies are given proportionally more weight [Borenstein M, Hedges L, Higgins J, Rothstein H. Introduction to Meta-Analysis. Chichester, West Sussex, UK: John Wiley & Sons; 2009]. Thus, through the use of a random effects model, the unique effect of each study was considered for the calculations. [Deeks JJ, Altman D, Bradburn M. Statistical methods for examining heterogeneity and combining results from several studies in metaanalysis. In: Egger M, Smith GD, Altman D, eds. Systematic Reviews in Health Care: MetaAnalysis in Context. London, UK: BMJ Books; 2001:285-312; Fleiss JL. The statistical basis of meta-analysis. Stat Methods Med Res. 1993;2:121-145].  Since, when testing the included studies data, we found statistical and epidemiological heterogeneity, therefore we used the random effects model. This allows to weigh more evenly the studies included in the meta-analysis. As a result smaller studies will have a greater relative impact on the combined total effect than fixed effect model [Sterne JA, Egger M, Moher D. Addressing reporting biases. In: Higgins JP, Green S, eds. Cochrane Handbook for Systematic Reviews of Interventions. Chichester, West Sussex, UK: John Wiley & Sons; 2008:297-333.].  Thus, we are able to assess the effect of anxiety in population, taking into account the fact that it can differ significantly between groups and represent a study-specific effect size. |

| **Reviewer 3** | **Changes by the authors** |
| --- | --- |
| The authors answered my questions, good effort. However the main limitation of this study remains to be the way the included studies measured the presence of anxiety. | Thank you very much for your comments.  Unfortunately, this problem is common for the vast majority of systematic reviews and meta-analyses. However, we tried to consider all possible factors underlying the heterogeneity of studies included in our review using random-effects and fixing-effects models. |
